# Emulsified Phosphatidylserine, Simple and Effective Peptide Carrier for Induction of Potent Epitope-Specific T Cell Responses

**DOI:** 10.1371/journal.pone.0060068

**Published:** 2013-03-22

**Authors:** Toru Ichihashi, Toshifumi Satoh, Chihiro Sugimoto, Kiichi Kajino

**Affiliations:** 1 Division of Collaboration and Education, Hokkaido University Research Center for Zoonosis Control, Sapporo, Japan; 2 Laboratory of Polymer Functional Chemistry, Faculty and Graduate School of Engineering, Hokkaido University, Sapporo, Japan; University of Rochester, United States of America

## Abstract

**Background:**

To induce potent epitope-specific T cell immunity by a peptide-based vaccine, epitope peptides must be delivered efficiently to antigen-presenting cells (APCs) *in vivo*. Therefore, selecting an appropriate peptide carrier is crucial for the development of an effective peptide vaccine. In this study, we explored new peptide carriers which show enhancement in cytotoxic T lymphocyte (CTL) induction capability.

**Methodology/Principal Findings:**

Data from an epitope-specific *in vivo* CTL assay revealed that phosphatidylserine (PS) has a potent adjuvant effect among candidate materials tested. Further analyses showed that PS-conjugated antigens were preferentially and efficiently captured by professional APCs, in particular, by CD11c^+^CD11b^+^MHCII^+^ conventional dendritic cells (cDCs) compared to multilamellar liposome-conjugates or unconjugated antigens. In addition, PS demonstrated the stimulatory capacity of peptide-specific helper T cells *in vivo*.

**Conclusions/Significance:**

This work indicates that PS is the easily preparable efficient carrier with a simple structure that delivers antigen to professional APCs effectively and induce both helper and cytotoxic T cell responses *in vivo*. Therefore, PS is a promising novel adjuvant for T cell-inducing peptide vaccines.

## Introduction

The immune system generates antibody-based humoral and T cell-based cellular immune responses to viruses, bacteria, and protozoa by recognizing a variety of components of the pathogen. Traditional vaccination strategies have successfully exploited these responses by utilizing whole, live attenuated or inactivated pathogens for the prevention of a large number of diseases; however, safety and production concerns have led to the development of non-infectious protein antigens or subunit vaccines [1]. Unlike live attenuated virus vaccines, which induce both humoral and cellular immunity, the weakness of protein antigens or subunit vaccines is that they only induce the antibody response, but not generally cellular (T cell) immunity.

Vaccines designed to induce epitope-specific CTL have been widely studied for many years. Among these, synthetic peptides have been shown to be a practical and useful vaccine since they are ultimately minimized T cell epitopes and would not be expected to induce harmful side effects. Furthermore, they can be produced easily [2], synthesized artificially, stored lyophilized at room temperature, and can be easily modified with functional chemical compounds. However, synthetic peptides have low immunogenicity, even when used in the combination with additional immunostimulators, e.g., Freund's adjuvants [3] or Toll-like receptor (TLR) stimulators. Therefore, we introduced a chemical modification to the antigenic peptide with the aim of enhancing the specific T cell response. In previous studies, we used multilamellar liposomes as a peptide carrier, which were conjugated with an immunogenic peptide on their surface. Consequently, the antigenicity of the peptide was significantly improved by liposome conjugation and induced potent specific CTL responses *in vivo*, which led to protection from lethal influenza virus infection in mice [4], [5]. Although liposome conjugation of the peptide was successful, several practical disadvantages of the liposome existed, including high production cost, low stability, and the difficulty of large-scale production. Therefore, a new peptide carrier that can enhance peptide immunogenicity by conjugation that is comparable to that observed with liposomes is required for practical vaccine usage.

It is commonly agreed that antigens of a pathogen should be actively introduced into professional APCs to induce a T cell response that is sufficient for clearance of infection. Internalized antigens are digested into short peptides, with a small portion of these then bindings to the major histocompatibility complex (MHC). T cells recognize these peptides presented by the MHC on the surface of APCs. Therefore, the primary role of a “peptide carrier” is to preferentially deliver as much antigen to the professional APCs as possible. If introduced without a carrier molecule, almost all of the peptide, particularly peptides that bind MHC class I, will be captured by cells other than APCs, because MHC class I exists ubiquitously in the body. Therefore, any candidate peptide carrier should be aggressively ingested by phagocytic APCs. Among the candidates, we selected several biological polymers, including chitosan [6], dendrimer [7], and carboxymethyl cellulose (CMC) [8], because they have already been assessed as carriers for drug-delivery systems and known to be ingested and degraded by macrophages. In addition, we also included hydrophilic phospholipids, i.e., components of the mammalian cell membrane, as phospholipid solution which consists of nano-sized micelles and/or unilamellar vesicles is easier to produce than multilamellar liposomes. In particular, phosphatidylserine (PS) is hydrophilic and exposed on the cell surface during the early stages of apoptosis [9], and thus functions as a target for phagocytosis by macrophages or dendritic cells [10], [11]. All of these molecules may facilitate antigen incorporation and presentation by professional APCs, and therefore may enhance induction of the antigen-specific T cell immune response.

Here, we have assessed several peptide carrier molecules with regards to their ability to activate CTL, and have further analyzed the functional properties of one promising candidate as an immuno-enhancer in comparison with liposomes.

## Materials and Methods

### Mice

C57BL/6 (B6) mice (6 weeks old; obtained from Japan SLC, Inc. (Hamamatsu, Japan)), and HLA-A24 transgenic (A24Tg) mice (kindly provided by Dr. François Lemonnier, Département d'Immunologie, Institut Pasteur, Paris, France) were bred under specific-pathogen-free conditions. A24Tg mice have a B6 background and express HLA-A*2402, and human β2 microglobulin and CD8 molecules, but do not express either murine H2D^b^ or H2K^b^. All experimental procedures were approved by the Hokkaido University Animal Care and Use Committee (approval number 10-0060), Sapporo, Japan.

### Peptides

Mouse MHC class I H-2K^b^-binding peptide OVA_257–264_ (SIINFEKL) and FITC-conjugated OVA_257–264_, H-2D^b^-binding peptide NP_366–374_ (ASNENVEAM) or NP_296–304_ (YSLVGIDPF) of influenza A virus A/HK483 (A/HongKong/483/97 [H5N1]), NP_366–374_ (ASNENTEAM) of influenza A virus A/PR8 (A/PuertoRico/8/34 [H1N1]), and human MHC class I HLA-A*2402-binding peptide NP_257–264_ (IFLARSALI) of A/HK483 virus and Tyrosinase_206–214_ (AFLPWHPLF) were obtained from Invitrogen (Carlsbad, CA).

Mouse MHC class II I-A^b^-binding peptide NP_311–325_ (QVYSLIRPNENPAHK) of A/PR8 virus [12] was obtained from MBL (Nagoya, Japan).

### Reagents

Dioleoylphosphatidylserine-Na (DOPS-Na) was purchased from Nippon Oil and Fat Corporation (Tokyo, Japan), and was dissolved in dH_2_O at 20 mM and sonicated for 6 min, then sterilized with a 0.22 µm filter (Nucleopore polycarbonate filter, Corning Costar, NY). The phosphatidylserine (PS) solution was stored in a nitrogen-filled tube at 4°C to prevent oxidation. 1-Ethyl-3-(3-dimethylaminopropyl) carbodiimide hydrochloride (EDC) was obtained from Thermo Fisher Scientific (Kanagawa, Japan). DQ™ ovalbumin (DQ-OVA), which exhibits green fluorescence upon proteolytic degradation, was purchased from Invitrogen. DAICHITOSAN FP was purchased from Dainichiseika Color & Chemicals (Tokyo, Japan). PAMPAM dendrimer G4-NH2 was purchased from Sigma-Aldrich Japan (Tokyo, Japan). To synthesize CMC, cellulose particles were reacted with sodium chloroacetate in isopropyl alcohol (IPA), and then the particles were washed by centrifugation.

### Conjugation of carrier materials to peptide or protein

For PS conjugation, at the final concentration of 10 mM PS solution, 0.1 M 2-morpholinoethanesulfonic acid (MES; pH 4.8), 1.25 mg/mL EDC and either 0.5 mM peptide solution, 100 µg/mL superfolder green fluorescent protein (sfGFP) or DQ-OVA were mixed in a rotator. The mixture was dialyzed in phosphate buffered saline (PBS). For conjugation of other carrier materials, 1 mg/mL chitosan, dendrimer or CMC solution was conjugated to each peptide using the same methods as described for the PS conjugation. To check the rate of peptide conjugation to the carrier material, 1 µM FITC-conjugated OVA_257–264_ peptide was added to each sample, and after dialysis (pH 7.0), the conjugation rate was measured using a Rotor Gene 6000 (Corbett Life Science, Australia). The coupling efficiency was about 85%.

### Liposomes

Liposomes consisting of dioleoylphosphatidyl choline, dioleoylphosphatidyl ethanolamine, dioleoylphosphatidyl glycerol acid, and cholesterol in a 4∶3∶2∶7 molar ratio were provided by Nippon Oil and Fat Corporation, and used as described previously [13]. The crude liposome solution was passed through a 0.22 µm membrane filter (Corning Costar). Liposome-conjugates were prepared using disuccinimidyl suberate (DSS) for cross-linking, as described previously [14].

### Cell culture

All cell cultures were performed in complete RPMI medium (RPMI-1640 medium (Invitrogen), 10% (v/v) FCS (Gibco), 50 µM 2-mercaptoethanol (Wako, Japan), 50 U/mL penicillin, and 50 µg/mL streptomycin (Nakalai Tesque, Kyoto, Japan)).

### *In vivo* cytotoxicity assay

Six- to 10-week-old B6 mice or A24Tg mice were immunized subcutaneously (s.c.) with carrier material-conjugated peptide or peptide without carrier (20 nmol/mouse) in the presence of poly(I:C) (10 µg/mouse; InvivoGen, San Diego, CA). For preparation of target cells, splenocytes from naïve B6 mice or A24Tg mice were suspended in PBS and then labeled with one of two concentrations (5 µM or 0.5 µM) of carboxyfluorescein diacetate succinimidyl ester (CFDA-SE, Invitrogen) at room temperature for 10 min. After the addition of equal volumes of heat-inactivated rabbit serum to quench the CFSE labeling reaction, cells were washed twice with PBS. Bright CFSE-labeled cells were pulsed with 0.5 µM peptide used for the immunization, on the other hand, dim CFSE-labeled cells were pulsed with an irrelevant peptide for 2 h at 37°C and 5% CO_2_. Five million cells cultured with respective peptides were mixed together and inoculated intravenously (i.v.) into mice which were immunized a week earlier. Twenty hours after target cells were inoculated, splenocytes were harvested, and CFSE-positive cells were analyzed by flow cytometry with dead cell exclusion performed by 7-aminoactinomycinD (7-AAD; Invitrogen) staining. NP_296–304_ or Tyrosinase_206–214_ was used as an irrelevant peptide. Reduction ratios of peptide-specific target cells were calculated using the following formula:

ITCR (inoculated target cell ratio)  =  (number of immunized peptide-pulsed cells harvested from PBS-injected mice)/(number of irrelevant peptide-pulsed cells harvested from PBS-injected mice), % specific reduction  =  {(number of irrelevant peptide-pulsed cells harvested from immunized mice) × ITCR – (number of immunized peptide-pulsed cells harvested from immunized mice)}/{(number of irrelevant peptide-pulsed cells harvested from immunized mice) × ITCR}×100.

### Cellular staining with MHC tetramer

Splenocytes from immunized mice with each PS-conjugated or unconjugated peptide in the presence of poly(I:C) were treated with anti-FcγRII/III mAbs (2.4G2) at 4°C for 20 min. After one wash in PBS, cells were stained with PE-conjugated H-2K^b^/OVA_257–264_ tetramer or PE-conjugated H-2D^b^/NP_366–374_ tetramer (MBL, Japan) at room temperature for 30 min, then stained with APC-conjugated anti-mouse CD8 mAb (clone: 53–6.7; BioLegend, San Diego, CA) at 4°C for 20 min. After two washes in PBS, cells were examined to quantify epitope-specific CTLs by flow cytometry. Dead cells were labeled with 7-AAD. Flow cytometric analyses were performed using a FACSCanto flow cytometer (BD Biosciences). Data are presented as dot plots using FlowJo software (Tree Star).

### Isolation of cells using a cell sorter

Splenocytes from B6 mice were treated with anti-FcγRII/III mAbs (2.4G2) and then stained with PE-conjugated anti-mouse CD11b mAb (clone: M1/70; eBioscience, San Diego, CA) and biotin-conjugated anti-mouse CD11c mAb (clone: N418; eBioscience) for 20 min at 4°C, followed by streptavidin-APC (Beckman Coulter, Fullerton, CA, USA) treatment for 20 min at 4°C. After two washes in PBS, dead cells were labeled with 7-AAD. Splenocytes were classified into five subpopulations based on the expression pattern of CD11b and CD11c. CD11b^−^CD11c^−^ cells, CD11b^int^CD11c^−^ cells, CD11b^high^CD11c^−^ cells, CD11b^+^CD11c^+^ cells and CD11b^−^CD11c^+^ cells were sorted by a MoFlo Astrios cell sorter (Beckman Coulter), resulting in cell purity of 85–99%.

### Analysis of antigen uptake and processing efficiency by PS conjugation

The five sorted cell populations were cultured with sfGFP, sfGFP-PS, sfGFP-liposome, DQ-OVA or DQ-OVA-PS (10 µg/mL each) for 60 min at 37°C. After the incubation, cells were washed with PBS, and then analyzed using a FACSCanto flow cytometer.

### Confocal laser scanning microscopy analysis

Splenocytes from B6 mice were treated with anti-FcγRII/III mAbs (2.4G2), then CD11b^+^ or CD11c^+^ cells were positively isolated using anti-mouse CD11b-conjugated or CD11c-conjugated MACS beads and LS columns (Miltenyi Biotec, Tokyo, Japan). Isolated cells were stained with PE-conjugated anti-mouse CD11b mAb (clone: M1/70) or biotin-conjugated anti-mouse CD11c mAb (clone: N418), followed by streptavidin-APC. After two washes in PBS, cells were cultured with sfGFP, sfGFP-PS, DQ-OVA, or DQ-OVA-PS (10 µg/mL each) and Hoechst33342 (2 µg/mL; Molecular Probes, Invitrogen) for 60 min at 37°C. After the incubation, cells were washed with PBS, then adhered onto a poly-L-lysine-coated glass bottom dish. Cells were analyzed using a LSM780 confocal laser scanning microscope system (Carl Zeiss).

### *In vitro* CD8^+^ T cells proliferation assay

CD8^+^ cells from the spleens of B6 mice immunized with PS-conjugated NP_366–374_ (A/PR8) peptide plus poly(I:C) were positively selected with BD Imag™ anti-mouse CD8 magnetic particles (BD Biosciences) to achieve >99% purity. For the preparation of stimulator cells, splenocytes were sorted according to five subpopulations based on the expression pattern of CD11b and CD11c using a MoFlo Astrios cell sorter. Sorted cells were incubated in the presence of CpG5002 (10 µM; Hokkaido System Science, Sapporo, Japan) for 2 h, then cultured with 10 µg/mL mitomycin C (MMC) for 1 h. After two washes, 2×10^4^ sorted cells were cultured with 2×10^5^ CD8^+^ cells from immunized mice for 2 days in a 96-well black plate (Corning Costar) at 37°C with 5% CO_2_ in 200 µL complete RPMI medium containing 0.1–100 nM PS-conjugated NP_366–374_ (A/PR8) peptide. Proliferation of NP_366–374_-pecific CD8^+^ cells was measured by Cell Proliferation ELISA, BrdU (chemiluminescence) kit (Roche, USA). Delta Relative Light Unit/second (Δrlu/s) was calculated using the following formula: Δrlu/s  =  (rlu/s of each peptide concentration) –(rlu/s of medium control).

### *In vitro* CD4^+^ T cells proliferation assay

CD4^+^ cells from the spleens of B6 mice immunized with PS-conjugated or unconjugated NP_311–325_ peptide in the presence of poly(I:C) were positively selected with BD Imag™ anti-mouse CD4 magnetic particles (BD Biosciences). Over 99% of the purified cells were CD4^+^ cells. Bone marrow cells were treated with recombinant murine granulocyte-macrophage colony-stimulating factor (rmGM-CSF) (R&D Systems, Minneapolis, MN USA) to stimulate differentiation into bone marrow-derived dendritic cells (BMDCs), which were used as a stimulator of CD4^+^ cells. BMDCs were cultured with 1 µg/mL lipopolysaccharide (LPS) (InvivoGen), 10 µg/mL functional grade purified anti-mouse CD40 (eBioscience) and 20 µg/mL anti-IL-10 (JES5-2A5) for 20 h at 37°C with 5% CO_2_, then cultured with 10 µg/mL MMC for 1 h. After two washes, MMC-treated activated BMDCs (1×10^4^) and CD4^+^ cells (1×10^5^) were co-cultured for 2 days in a 96-well black plate (Corning Costar) at 37°C with 5% CO_2_ in 200 µL complete RPMI medium containing 0.01–10 µM NP_311–325_ peptide. Proliferation of NP_311–325_-specific CD4^+^ cells was measured as above.

### Statistical analyses

Statistical analyses were carried out using the Student's t-test, and multiple comparison analysis was performed using the Tukey-Kramer method. P values<0.05 were considered significant.

## Results

### PS-conjugated peptide significantly enhances peptide-specific cytotoxic activity *in vivo*

**Figure 1:**
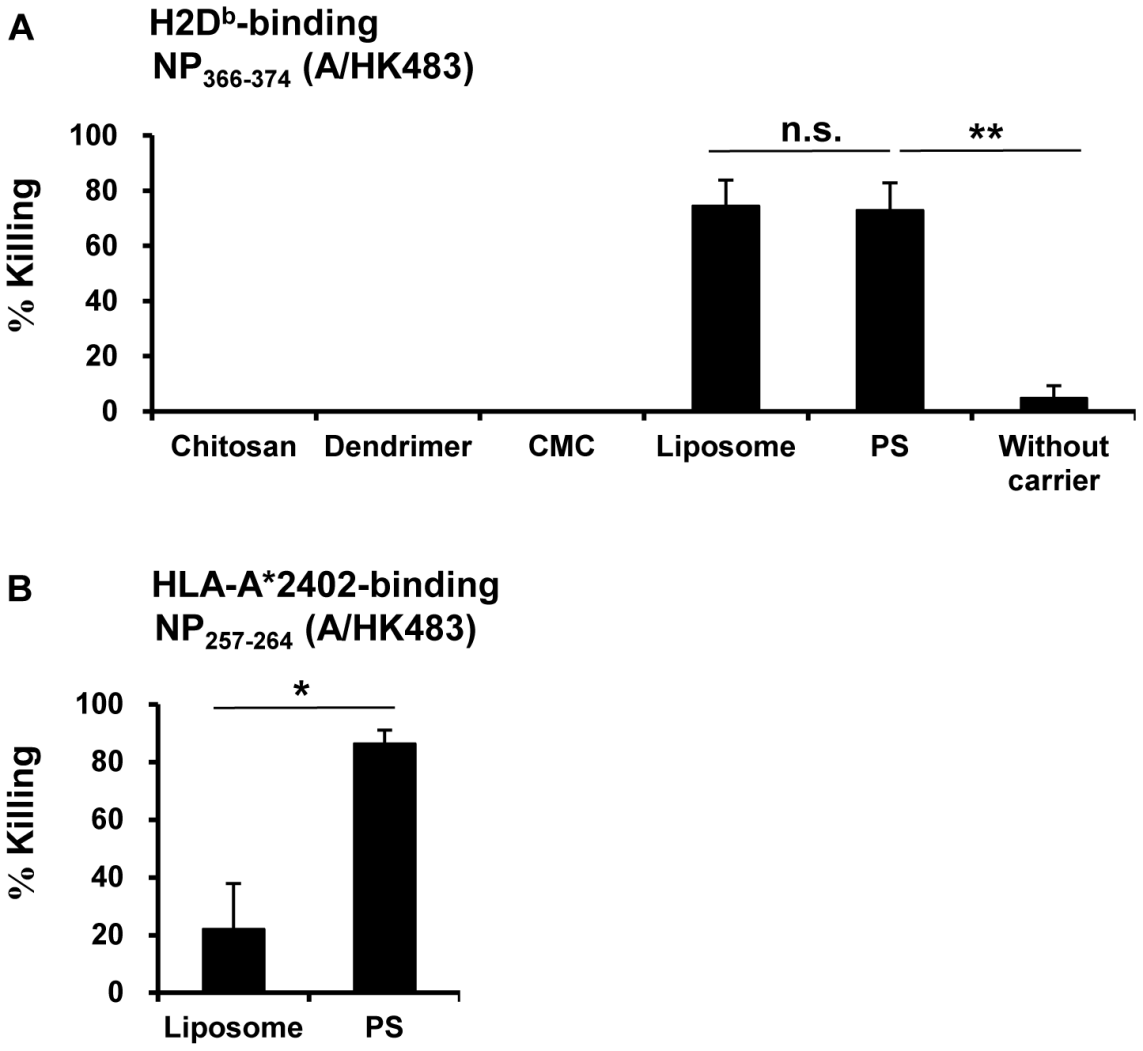
Mice immunized with PS-conjugated peptide induced epitope-specific CTL effectively *in vivo*. (A) B6 mice (3 to 4 mice per group) were immunized s.c. with each carrier-conjugated NP_366–374_ (A/HK483) peptide or peptide without carrier in the presence of poly(I:C). Seven days after the immunization, bright CFSE-labeled target cells pulsed with peptide used for the immunization and dim CFSE-labeled target cells pulsed with an irrelevant peptide were injected i.v. as an *in vivo* cytotoxicity assay. Viability of the target cells in the spleen was examined 20 h after injection. Reduction ratios of epitope-specific target cells were calculated using the formula described in Materials and Methods. (B) A24Tg mice (3 mice per group) were inoculated with PS- or liposome-conjugated NP_257–264_ (A/HK483) peptide. The *in vivo* cytotoxicity assay was performed as described for Figure 1A. n.s. indicates not significant. *p<0.01, **p<0.0001.

**Figure 2:**
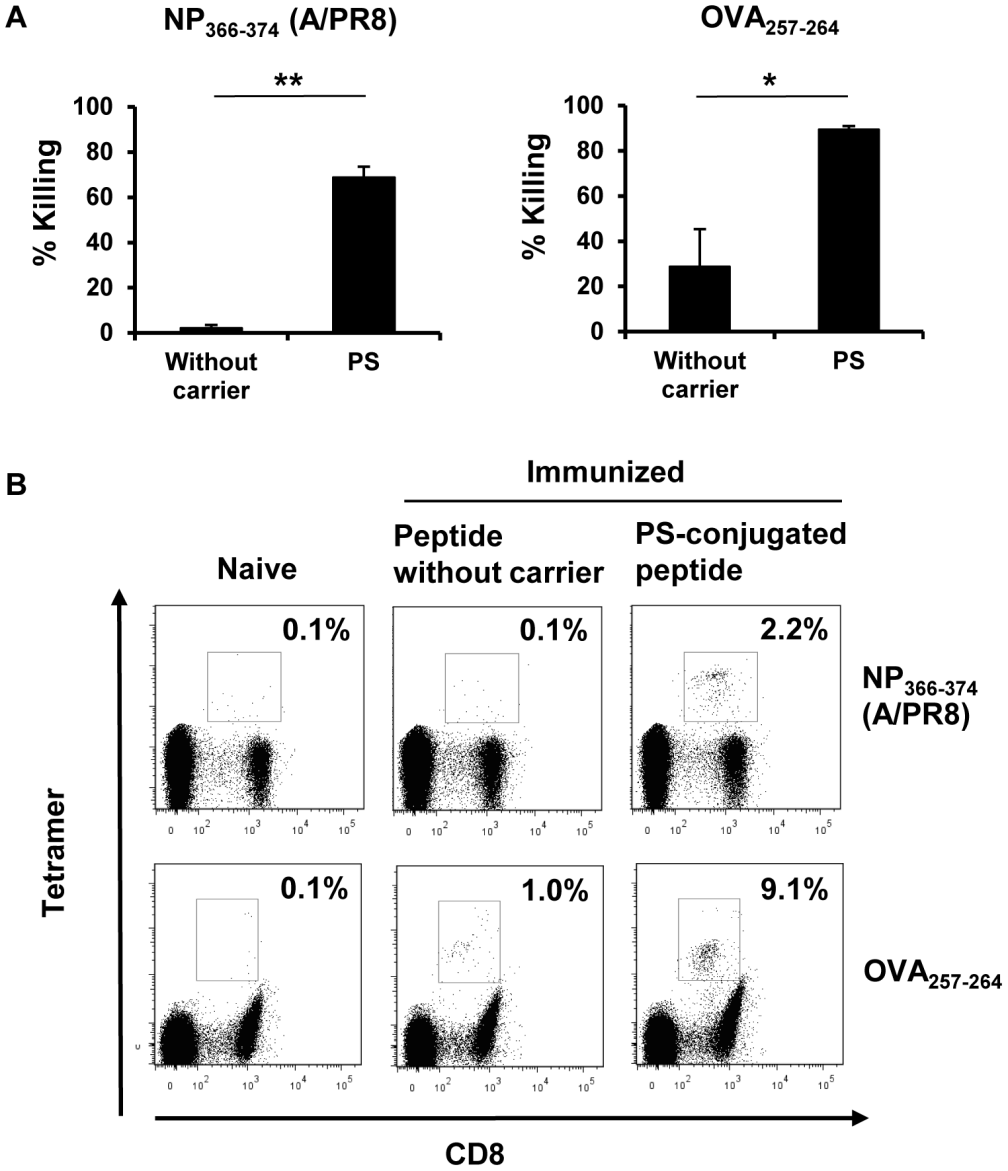
Frequency of epitope-specific CD8^+^ T cells in immunized mice. (A) PS-conjugated NP_366–374_ (A/PR8) peptide, PS-conjugated OVA_257–264_ peptide or unconjugated peptide was inoculated into B6 mice (3 to 4 mice per group). The *in vivo* cytotoxicity assay was performed as described in Figure 1. *p<0.01, **p<0.0001. (B) Splenocytes from naïve mice and mice immunized with PS-conjugated or unconjugated peptide in the presence of poly(I:C) were stained with tetramer and anti-mouse CD8 Ab. The percentage indicates the tetramer-positive cells in total CD8^+^ cells. The experiment was repeated three times with similar results.

To identify which carrier is most effective for peptide-specific CTL induction *in vivo*, B6 mice were immunized s.c. with carrier-conjugated or unconjugated antigen peptide in the presence of poly(I:C). *In vivo* cytotoxicity assay data showed that NP_366–374_ (A/HK483) peptide conjugated with chitosan, dendrimer, or CMC did not induce epitope-specific CTL at all, which was less than unconjugated peptide alone. In contrast, PS-conjugated peptide induced epitope-specific killing effectively *in vivo* compared to that induced by unconjugated peptide. Furthermore, the induction efficiency of peptide-specific killing by PS-conjugated peptide was comparable to (Figure 1A) or significantly higher than (Figure 1B) those of liposome-conjugates. Enhancement of the CTL induction effect by PS was confirmed by NP_366–374_ from another influenza virus strain (A/PR8) or OVA_257–264_ (Figure 2A). Thus, it was clearly demonstrated that enhancement of peptide-specific target cell killing by PS conjugation did not depend on the peptide sequences. Additionally, we also examined the influence of vaccination route and particle size on the CTL induction capability of PS- and liposome-conjugated peptides. When given to mice via the i.v., the PS-conjugated peptide was able to induce epitope-specific killing effectively; however, by the same route of administration, the CTL induction efficiency by liposome-conjugated peptide was very low (Figure S1). Moreover, the average size of PS in solution was around half that of the liposomes (Table S1).

### The epitope-specific CTL population was expanded by immunizing with PS-conjugated peptide

We next investigated the frequency of the epitope-specific CTL population in the spleens of mice which were immunized with PS-conjugated peptide. CD8^+^ splenocytes expressing peptide antigen-specific T cell receptors detected by H-2D^b^/NP_366–374_-tetramer or H-2K^b^/OVA_257–264_-tetramer were analyzed 7 days after s.c. immunization with PS-conjugated or unconjugated peptides. As expected, the population of tetramer^+^ CD8^+^ CTL was increased in mice immunized with NP_366–374_ -PS (2.2%) or OVA_257–264_-PS (9.1%), compared to mice immunized with unconjugated NP_366–374_ (0.1%) or OVA_257–264_ (1.0%), respectively (Figure 2B). Moreover, the data from the cytotoxicity analysis (Figure 2A) and those from the frequency analysis of epitope-specific CTL population (Figure 2B) correlated well with each other. These findings suggest that PS conjugation strongly promotes antigen-specific CTL expansion and activation induced by peptide immunization.

### Professional APCs play a predominant role in the uptake and digestion of the PS-antigen complex

**Figure 3:**
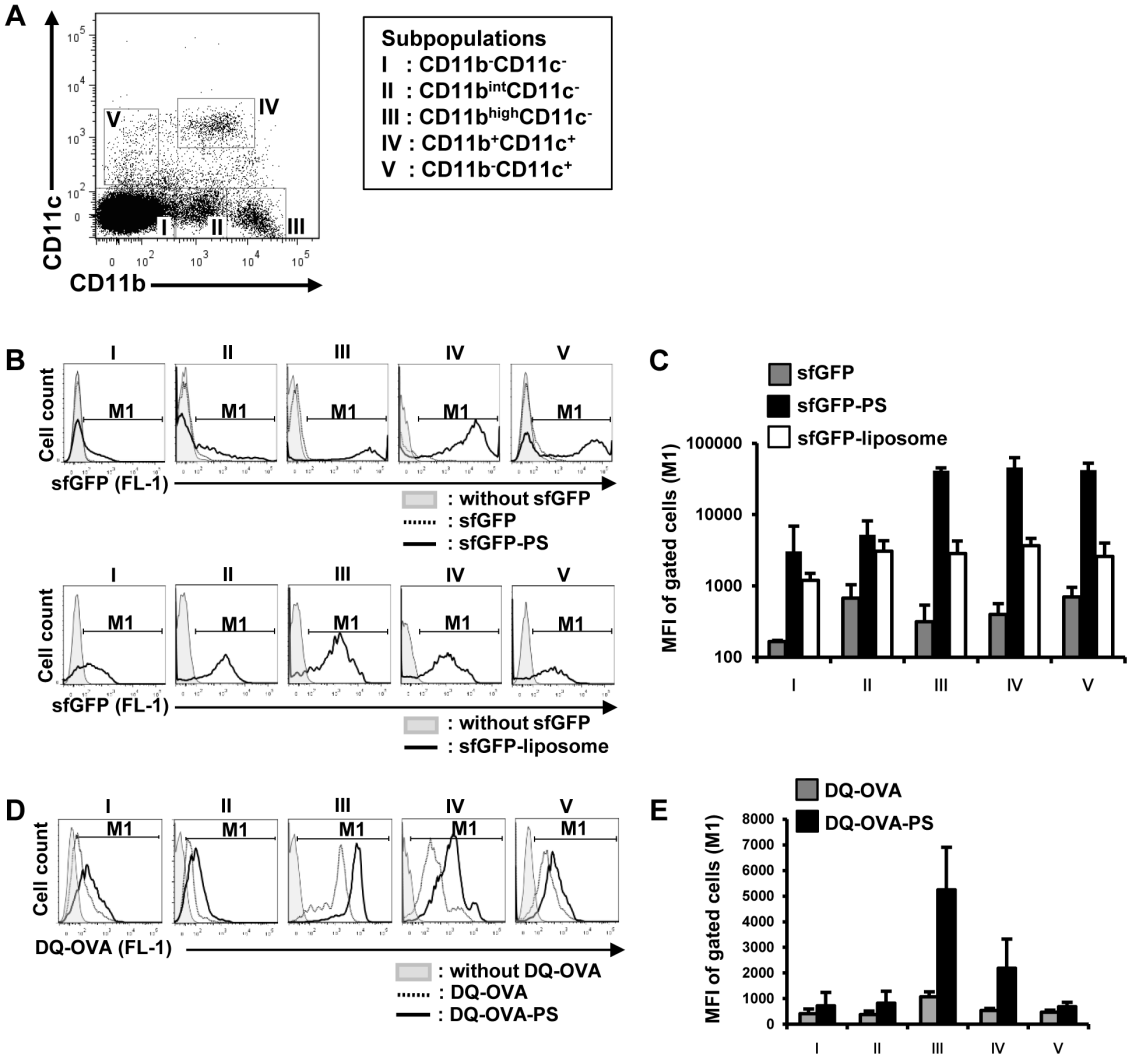
PS-conjugated Ags were captured by professional APCs effectively. (A) Splenocytes were classified into five subpopulations (I to V) based on the expression pattern of CD11b and CD11c. I:CD11b^−^CD11c^−^ cells, II:CD11b^int^CD11c^−^ cells, III:CD11b^high^CD11c^−^ cells, IV:CD11b^+^CD11c^+^ cells, V:CD11b^−^CD11c^+^ cells. (B, C) Each isolated population was co-cultured with sfGFP, sfGFP-PS or sfGFP-liposome for 60 min, and then the amount of uptake was analyzed by flow cytometry. (D, E) Each population of isolated cells was co-cultured with DQ-OVA or DQ-OVA-PS for 60 min, and then the efficiency of antigen degradation processing was analyzed by flow cytometry.

To investigate the mechanism underlying the enhancement of CTL induction by PS as a peptide carrier, we analyzed the PS particle incorporation into cells after incubation with several fractionated splenocyte populations. To differentiate professional APCs, we classified splenocytes into five subpopulations based on the expression pattern of CD11b and CD11c [15]–[17] and isolated them by cell sorting (Figure 3A). Analysis of cell surface markers (Figure S2) indicated that the CD11b^−^CD11c^−^ population I consisted mainly of T- and B-lymphocytes [18], [19], the CD11b^int^CD11c^−^ population II consisted mainly of NK cells [20], and the CD11b^high^CD11c^−^ population III consisted of macrophages and granulocytes [21], [22]. Furthermore, the CD11b^+^CD11c^+^ population IV consisted of conventional DCs (cDCs) [23] and the CD11b^−^CD11c^+^ population V contained plasmacytoid DCs (pDCs) [24]. After the incubation of these five populations with sfGFP, PS-conjugated sfGFP (sfGFP-PS) or liposome-conjugated sfGFP (sfGFP-liposome), flow cytometric analysis was performed. Although sfGFP-liposomes were captured equally among these five populations, the uptake of sfGFP-PS was increased significantly in CD11b^high^CD11c^−^ cells, CD11b^+^CD11c^+^ cells, and CD11b^−^CD11c^+^ cells (Figure 3B, C). These results indicated that PS-conjugated antigen was more incorporated into professional APCs than liposome-conjugated antigens. Furthermore, to analyze the efficiency of antigen processing by PS conjugation, each cell population was cultured with DQ-OVA or PS-conjugated DQ-OVA (DQ-OVA-PS), because DQ-OVA becomes fluorescent only after degradation of OVA protein. After one hour, flow cytometric analysis showed that CD11b^high^CD11c^−^ and CD11b^+^CD11c^+^ cells captured and degraded DQ-OVA more efficiently by PS conjugation than other subpopulations (Figure 3 D, E).

### Confocal laser scanning microscopy analysis of Ag-captured cells

**Figure 4:**
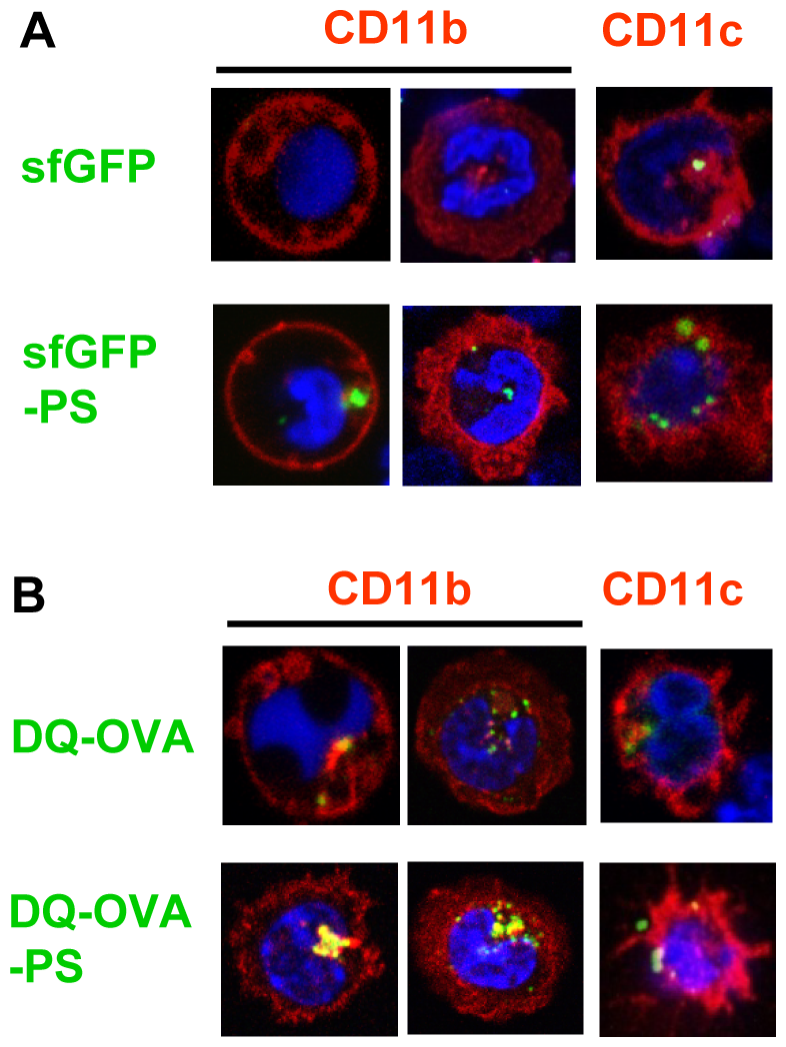
Confocal laser scanning microscopy analysis of splenocytes co-cultured with PS-conjugated antigens. (A, B) CD11b^+^ or CD11c^+^ cells were cultured with sfGFP, sfGFP-PS, DQ-OVA or DQ-OVA-PS plus Hoechst33342 for 60 min at 37°C. After the incubation, cells were washed with PBS, and then analyzed under a LSM780 confocal laser scanning microscope system. Blue: cell nucleus, Green: sfGFP or DQ-OVA, Red: CD11b or CD11c.

We performed morphological analysis to confirm that PS-conjugated antigens were internalized and digested in the CD11b^+^ or CD11c^+^ cells after the ligation to the PS receptor. In both CD11b^+^ and CD11c^+^ cells, sfGFP was observed as spots surrounded by plasmatic membrane, indicating that ingested antigens were internalized and accumulated in the phagosomes of these cells. Time course analyses showed that these spots gathered into a cluster over time (Movie S1). Consistent with the results of flow cytometric analysis (Figure 3), more spots were observed inside the cells pulsed with sfGFP-PS (Figure 4A). Similarly, DQ-OVA-PS-pulsed cells showed more and larger spots than cells cultured with DQ-OVA (Figure 4B). These findings suggest that antigen processing is increased because of PS-induced enhanced protein uptake by APCs.

### Cell subsets as potent inducers for antigen-specific CTL

**Figure 5:**
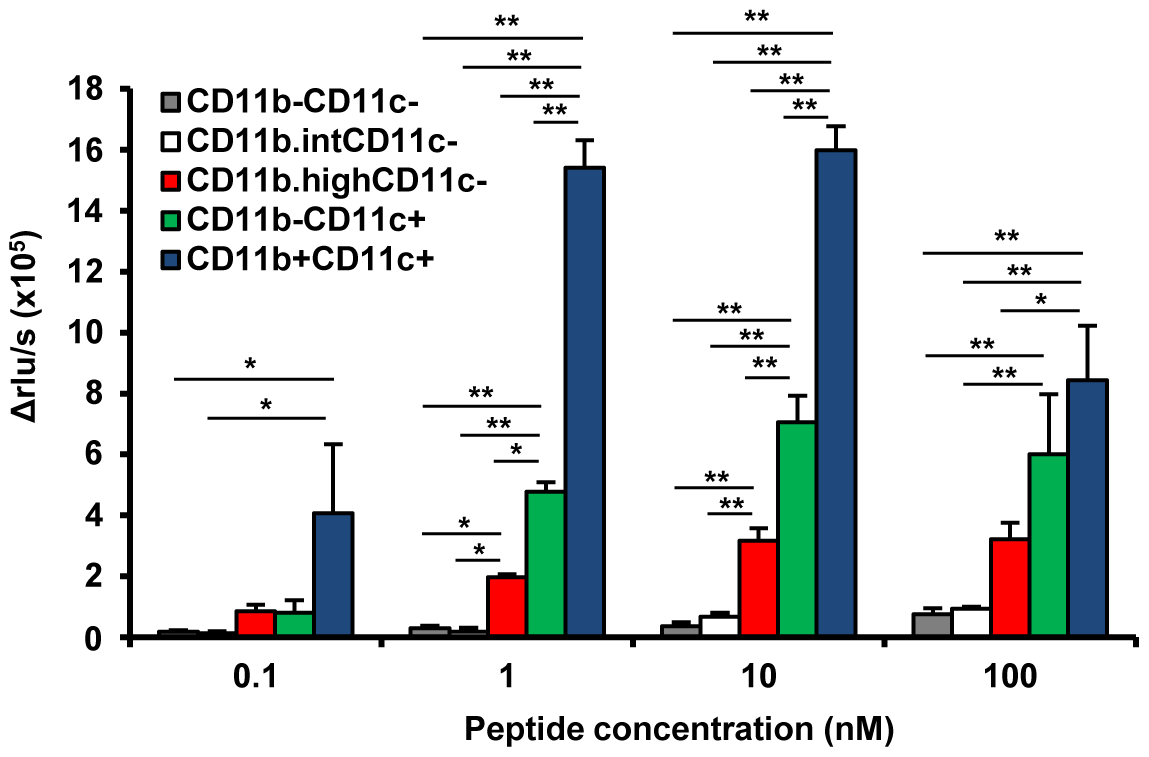
CD11c^+^CD11b^+^ cells are main APCs in peptide vaccines. The five kinds of sorted splenocytes were activated with CpG5002 *in vitro* and cultured with CD8^+^ T cells from NP_366–374_-PS immunized mice with serial dilutions of PS-conjugated NP_366–374_ peptide for 2 days. Proliferation of NP_366–374_-specific CD8^+^ cells was measured by BrdU uptake. The experiment was repeated twice with similar results. *p<0.05, **p<0.01.

To analyze the antigen-presenting capacity of each cell population, CD8^+^ T cells specific for NP_366–374_ peptide were co-cultured with each splenocyte population purified by flow cytometric sorting using serial dilutions of PS-conjugated NP_366–374_ peptide for 2 days. *In vitro* T cell proliferation assay demonstrated that PS-conjugated peptides were presented to T cells by CD11b^high^CD11c^−^ cells and CD11c^+^ cells, with CD11c^+^CD11b^+^ cells having maximum efficiency (Figure 5). Population analysis (Figure S2) revealed that the main APCs to stimulate epitope-specific CTL were cDCs. Thus, cDCs are likely to be the main target of the PS-conjugated vaccine.

### PS-conjugated MHC class II-restricted peptide induces epitope-specific T helper cells effectively *in vivo*

Cellular immune responses, consisting of both CD8^+^ CTL and CD4^+^ T helper (Th) cells, play an essential role in the control of viral infection. The antigen processing and presentation pathway of MHC class II molecules differs to that of MHC class I molecules. In general, extracellular antigens are presented as 15–17 mer epitopes via MHC class II molecules to CD4^+^ Th cells.

**Figure 6:**
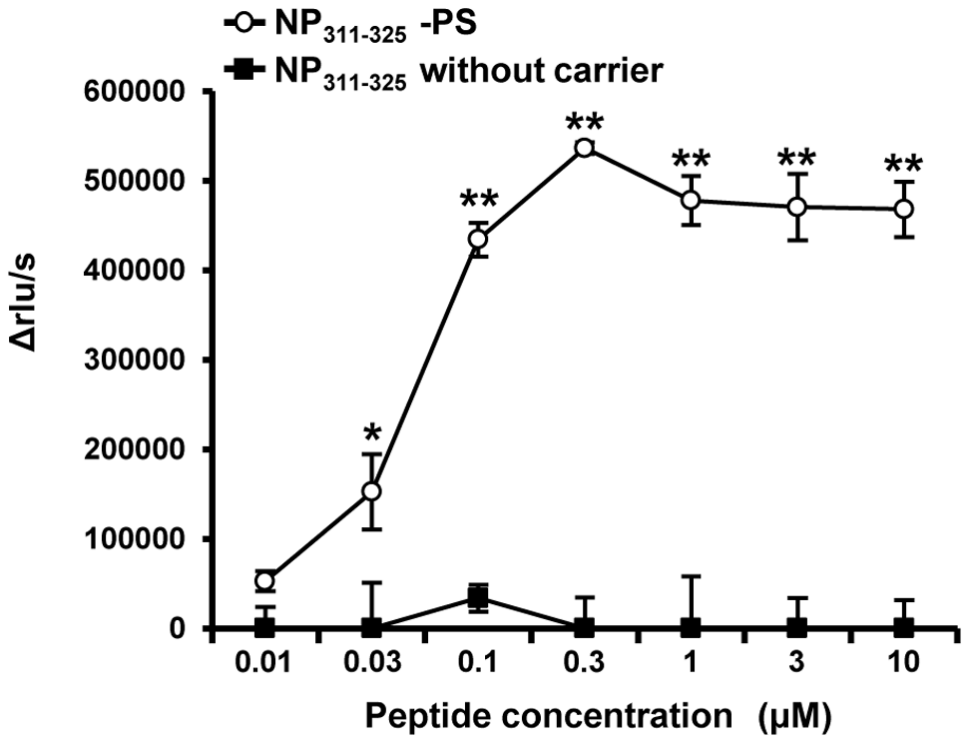
Proliferation assay of epitope-specific helper T cells. CD4^+^ T cells from mice immunized with PS-conjugated or unconjugated peptide were co-cultured with activated BMDCs for 2 days in complete RPMI medium containing the indicated concentration of NP_311–325_ peptide. Proliferation of NP_311–325_-specific CD4^+^ Th cells was measured by BrdU uptake. The experiment was repeated three times with similar results. *p<0.05, **p<0.005

We examined whether PS conjugation can effectively induce epitope-specific CD4^+^ Th cells *in vivo* by immunization of MHC class II-restricted epitopes. B6 mice were immunized s.c. with PS-conjugated or unconjugated NP_311–325_ peptide in the presence of poly(I:C). CD4^+^ cells in the spleens from immunized mice were collected 7 days after the vaccination, and proliferation of NP_311–325_-specific CD4^+^ Th cells was measured by BrdU uptake. The proliferative response of epitope-specific Th cells in mice immunized with PS-conjugated peptide was higher than that in mice immunized with unconjugated peptide (Figure 6). This result indicates that PS-conjugated peptide can more effectively induce epitope-specific T cells (both CTL and Th cells) compared to immunization with peptide alone.

## Discussion

For many years, lipid-based carriers have been investigated for potential applications in drug, DNA, peptide, and protein delivery [25], [26]. In particular, liposome and lipid nano-particles have been studied for practical use in humans. Consistent with these studies, the screening result of peptide carriers indicated that PS was a potent enhancer of not only the peptide-induced CTL response, but also of the Th cell response. PS is normally confined to the inner lipid bilayer of the cell membrane, and is immediately exposed to the cell surface when cells initiate apoptosis [9]. A variety of cell surface molecules [27]–[30] have been implicated in the recognition of apoptotic cells. Among them, PS is known to trigger the specific recognition and removal of apoptotic cells by its receptors Tim4 and Tim1, which function in the engulfment of apoptotic cells and in intracellular signaling of macrophages and dendritic cells [31]. Therefore, it might be expected that PS conjugation would disguise the peptide as an antigen epitope from apoptotic cells and lead to enhanced recognition of the epitope by professional APCs, thereby resulting in a potent induction of the immune response.

On the other hand, chitosan has been also widely used in vaccination formulations because of its ability to enhance immunogenicity [32]–[37]. However, in contrast to previous studies, chitosan-conjugated peptide did not induce epitope-specific CTL *in vivo*, even though the peptide without a carrier was able to induce a weak CTL response. Similarly, CTL inhibition by dendrimer or CMC conjugation was also observed. It is assumed that these molecules utilize similar mechanisms of immune modulation as chitosan, so the detailed inhibition mechanism of these molecules requires further investigation and clarification.

Enhanced lymphatic penetration and retention are also considered to be improvements in the drug-delivery system mediated by lipid-based carriers [38]. Likewise, the improvements of this PS carrier are thought to consist of two factors, i.e., its nanoscale size and its anionic charge. Compared to the liposome we had used previously, PS formed a smaller-sized structure in water, which is expected to be an advantage in terms of tissue penetration after administration. In addition, we compared several phosphatidyl-lipids to determine whether the serine residue of PS was significant for its activity. Among them, dioleoylphosphatidyl glycerol (DOPG) also induced a certain level of *in vivo* cytotoxic activity compared to the liposome-conjugated peptide. In contrast, the CTL induction efficiency by dioleoylphosphatidyl ethanolamine (DOPE)-conjugated peptide was in the same range as that of unconjugated or oleoyl micelle-conjugated peptide (data not shown). The remarkable common feature of the DOPS and DOPG, but not of the DOPE or oleoyl micelles, is that the surface is negatively charged. A recent whole-body fluorescence imaging study revealed that the anionic nature of the polymer contributed to the enhanced lymphatic uptake and the prolonged deep-nodal retention [39]. Therefore, it is likely that the anionic PS particles preferentially migrate to lymph nodes (LNs) via lymphatic vessels and remain there, and consequently, they are able to transport cross-linked antigen peptides to LN-resident DCs efficiently.

Although liposome-conjugated peptide was comparable to PS-conjugated peptide in inducing antigen-specific CTL, the distribution patterns among the 5 kinds of sorted populations of ingested liposomes are considerably different from those of PS. The *in vitro* GFP tracking experiment (Figure 3B, C) showed that liposome intake was not specific for cell populations; however, PS was mainly incorporated into professional APCs. Furthermore, the amount of PS-conjugate taken up by APCs was more than that of liposomes, as determined by FACS analyses.

This difference of PS and liposome in the interaction with APCs may be explained by the following facts. Previously, Tanaka *et al.* reported that antigens coupled to oleoyl liposomes might be taken up by penetration and/or pinocytosis [40]. Presumably, liposome incorporation into cells depends on non-specific fusion of cell membrane mimics. On the other hand, PS incorporation by APCs is thought to be mainly through receptor specific [41], and slight receptor-independent phagocytosis by feature of PS which has poor binding to cells [42], because APCs preferentially absorbed PS-conjugated GFP despite the size of PS being smaller than that of the liposome. Therefore, we propose that receptor-specific active incorporation of antigen peptide to professional APCs, in particular, DCs in regional LNs, is enhanced by PS conjugation and leads to increased accumulation of antigen epitope, and subsequently, increased induction of peptide sequence-specific CTL activity *in vivo*.

Moreover a remarkable difference between PS and liposome was observed. PS-conjugated peptide but not liposome-conjugated peptide could elicit peptide-specific CTL *in vivo* after i.v. administration. The percentage of APCs, especially DCs, in blood is very low compared to that in tissues such as subcutis, liver and lung alveoli, so it is difficult for antigen peptide to reach DCs efficiently when administrated i.v.. Incorporation of liposome was observed regardless of cell populations, while PS was incorporated into APCs preferentially in *in vitro* experiments. From these results, it is considered that when liposome was administrated i.v., most of liposome was trapped by blood cells before reaching DCs. In contrast, PS is considered to be able to reach DCs efficiently without trapping in the blood, so that it is able to induce epitope-specific CTL even when administrated i.v.

Considering the simplicity of its preparation, PS is a promising material because it has a hydrophillic terminus and easily forms nano-sized structures in water after several minutes of sonication. While liposomes require organic solvents during their preparation, and a large amount of time and energy input to produce the multilamellar form, the preparation of PS solution is comparatively simple and does not require organic solvents, and therefore, PS meets this essential requirement for application to vaccines.

In conclusion, we have developed a novel peptide carrier that is able to enhance peptide-specific cytotoxic and/or helper T cell responses effectively. The enhancing mechanism is likely due to the efficient and preferential transportation of antigen peptides to regional LN-resident DCs, through mainly the apoptotic cell receptor-dependent endocytosis, and the ability to penetrate the tissues and lymphatics. With its ease of preparation, PS is a promising carrier candidate for antigen peptides as a component of T cell immunity-inducing vaccines.

## Supporting Information

Figure S1 Mice immunized intravenously with PS-conjugated peptide were able to induce epitope-specific CTL.B6 mice (3 to 4 mice per group) were immunized s.c. or i.v. with PS- or liposome-conjugated NP_366–374_ (A/HK483) peptide in the presence of poly(I:C). Seven days after the immunization, bright CFSE-labeled target cells pulsed with peptide used for the immunization and dim CFSE-labeled target cells pulsed with an irrelevant peptide were injected i.v. as an *in vivo* cytotoxicity assay. Viability of the target cells in the spleen was examined 20 h after injection. Reduction ratios of epitope-specific target cells were calculated using the formula described in Materials and Methods.
(TIF)

Figure S2 Population analysis of five subpopulations based on CD11b and CD11c expression patterns.Cell surface markers of each subpopulation were analyzed by flow cytometry: CD3 is a T cell marker; CD19 is a B cell marker; B220 is a marker of B cells and a subset of NK cells; CD49b is an NK cell marker; F4/80 is a marker of monocytes, macrophages and a subset of dendritic cells; Gr-1 is a granulocyte marker; MHCII is expressed on professional antigen-presenting cells.
(TIF)

Table S1 Particle size of PS and liposomes.
(DOCX)

File S1 Supplementary Materials and Methods.
(DOCX)

Movie S1 Supplemental movie.
(MOV)
