# Supplementary figures and images for: Emulsified Phosphatidylserine, Simple and Effective Peptide Carrier for Induction of Potent Epitope-Specific T Cell Responses
Source: PLoS One. 2013 Mar 22;8(3):e60068. doi: 10.1371/journal.pone.0060068 (PMC3606214; doi:10.1371/journal.pone.0060068)

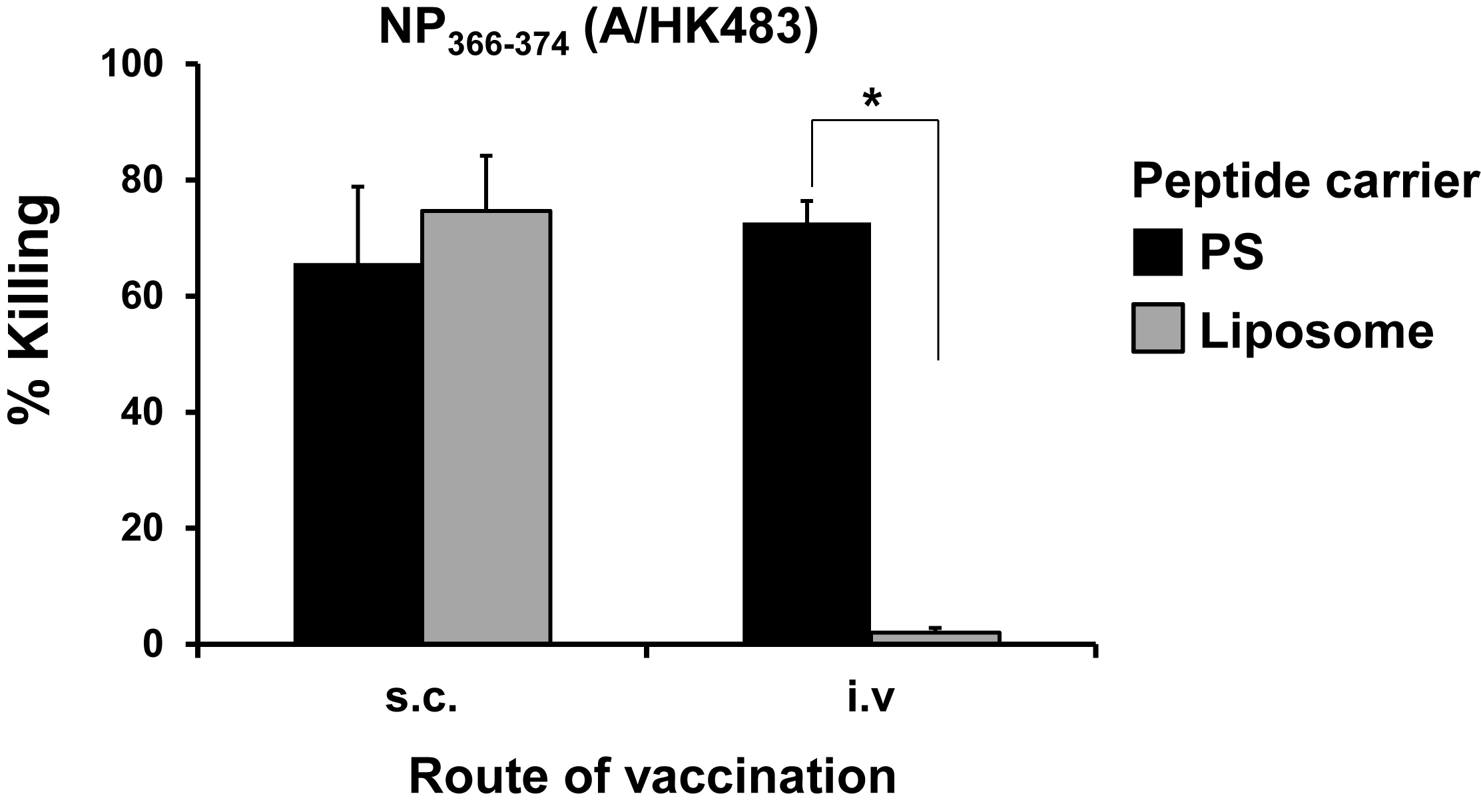

Supplement: Figure S1 — Mice immunized intravenously with PS-conjugated peptide were able to induce epitope-specific CTL.B6 mice (3 to 4 mice per group) were immunized s.c. or i.v. with PS- or liposome-conjugated NP366–374 (A/HK483) peptide in the presence of poly(I:C). Seven days after the immunization, bright CFSE-labeled target cells pulsed with peptide used for the immunization and dim CFSE-labeled target cells pulsed with an irrelevant peptide were injected i.v. as an in vivo cytotoxicity assay. Viability of the target cells in the spleen was examined 20 h after injection. Reduction ratios of epitope-specific target cells were calculated using the formula described in Materials and Methods. (TIF) [file pone.0060068.s001.tif]

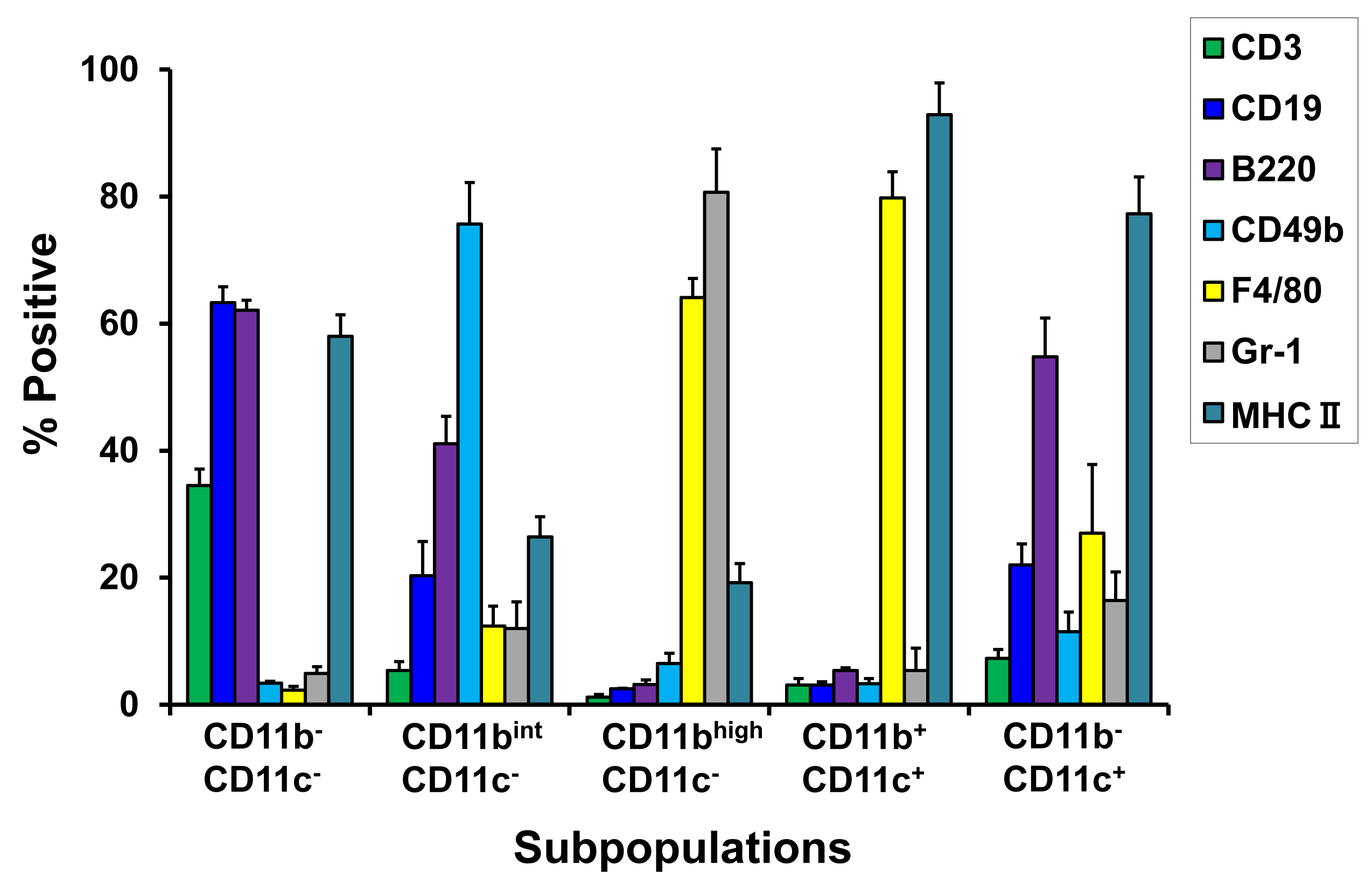

Supplement: Figure S2 — Population analysis of five subpopulations based on CD11b and CD11c expression patterns.Cell surface markers of each subpopulation were analyzed by flow cytometry: CD3 is a T cell marker; CD19 is a B cell marker; B220 is a marker of B cells and a subset of NK cells; CD49b is an NK cell marker; F4/80 is a marker of monocytes, macrophages and a subset of dendritic cells; Gr-1 is a granulocyte marker; MHCII is expressed on professional antigen-presenting cells. (TIF) [file pone.0060068.s002.tif]
