# Supplementary material for: Emulsified Phosphatidylserine, Simple and Effective Peptide Carrier for Induction of Potent Epitope-Specific T Cell Responses
Source: PLoS One. 2013 Mar 22;8(3):e60068. doi: 10.1371/journal.pone.0060068 (PMC3606214; doi:10.1371/journal.pone.0060068)
Supplement: Table S1 — Particle size of PS and liposomes. (DOCX) [file pone.0060068.s003.docx]

**Table S1. Particle size of PS and liposomes**

|  | Average size | (size range) |
| --- | --- | --- |
| PS (10 mM) | 85.8 nm | (28.8-122.2 nm) |
| Liposomes (16 mM) | 192.1 nm | (44.7-255.8 nm) |
