# Supplementary material for: Emulsified Phosphatidylserine, Simple and Effective Peptide Carrier for Induction of Potent Epitope-Specific T Cell Responses
Source: PLoS One. 2013 Mar 22;8(3):e60068. doi: 10.1371/journal.pone.0060068 (PMC3606214; doi:10.1371/journal.pone.0060068)
Supplement: File S1 — Supplementary Materials and Methods. (DOCX) [file pone.0060068.s004.docx]

**Supplementary Materials and Methods**

**Immunization with PS-conjugated peptide by different administration routes**

Six- to 10-week-old B6 mice were immunized s.c. or i.v. with PS-conjugated peptide or liposome-conjugated peptide in the presence of poly(I:C) (10 μg/mouse; InvivoGen). Splenocytes from naïve B6 mice were suspended in PBS and then labeled with one of two concentrations (5 μM or 0.5 μM) of CFDA-SE (Invitrogen) at room temperature for 10 min. After the addition of equal volumes of heat-inactivated rabbit serum to quench the CFSE-labeling reaction, cells were washed twice with PBS. Bright CFSE-labeled cells were pulsed with 0.5 μM peptide used for the immunization, on the other hand, dim CFSE-labeled cells were pulsed with an irrelevant peptide for 2 h at 37°C and 5% CO_2_. Five million cells cultured with respective peptides were mixed together and inoculated i.v. into mice which were immunized a week earlier. Twenty hours after target cells were inoculated, splenocytes were harvested, and CFSE-positive cells were analyzed by flow cytometry with dead cell exclusion performed using 7-AAD (Invitrogen) staining. Reduction ratios of epitope-specific target cells were calculated using the formula described in Materials and Methods.

**Analysis of five subpopulations**

Five subpopulations based on the CD11b and CD11c expression patterns were stained with FITC-conjugated anti-mouse Ly-6G/Ly-6C (Gr-1) mAb (clone: RB6-8C5, BioLegend), anti-mouse F4/80 mAb (clone: BM8, BioLegend), anti-mouse B220 mAb (clone: RA3-6B2, eBioscience), anti-mouse CD3e mAb (clone: 145-2C11, BioLegend), anti-mouse CD19 mAb (clone: 6D5, BioLegend), anti-mouse CD49b mAb (clone: DX5, BioLegend), anti-mouse MHC class II (I-A/I-E) mAb (clone: M5/114.15.2 , BioLegend) for 20 min at 4°C. After two washes in PBS, dead cells were labeled with 7-AAD, and then cells were analyzed using a FACSCanto flow cytometer (BD Biosciences).

**Measurement of particle size of PS and liposomes**

Average particle size of PS in solution which was sonicated and passed through a filter and multilamelar liposomes was measured using a Fiber Dynamic Light Scattering Spectrophotometer (FDLS-3000; Otsuka Electronics, Japan).

**Moving image of PS-conjugated sfGFP uptake by CD11c^+^ cells**

CD11c^+^ cells purified by MACS column were cultured with sfGFP-PS and a moving image of the uptake was analyzed under a LSM780 confocal laser scanning microscope system.
